# The psychological impact of the COVID emergency on Italian nursing homes staff and the effectiveness of eye movement desensitization and reprocessing

**DOI:** 10.3389/fpsyg.2022.969028

**Published:** 2022-10-12

**Authors:** Elisa Faretta, Giada Maslovaric, M. Ignazia Garau, Gabriella Marmondi, Laura Piras, Simona Rezzola, Alessia Incerti, Anna Nardoni, Marco Pagani, Eugenio Gallina

**Affiliations:** ^1^EMDR Italy Association, Varedo, Italy; ^2^Institute of Cognitive Sciences and Technologies, Consiglio Nazionale delle Ricerche, Rome, Italy; ^3^Centro di Ricerca e Studi in Psicotraumatologia, Milan, Italy

**Keywords:** nursing home, EMDR, PTSD, COVID-19, Italy

## Abstract

Residential nursing homes were particularly badly affected by the first wave of COVID-19, with large numbers of their frail person getting infected with COVID-19 and dying. The staff in these structures were catapulted into a reality very different from what they were used to. They had to adapt the way they used to take care of their patients in a very short space of time and in a scenario that was continually changing. In this manuscript we describe the subjective experience of staff in a number of Italian nursing homes during the first wave of the COVID-19 pandemic; and we report data showing the effectiveness of the Eye Movement Desensitization and Reprocessing (EMDR) treatment provided to support them during this Pandemic.

## Introduction

The COVID-19 emergency has been a mass and humanitarian disaster and we are still only able to gauge the tip of the iceberg in terms of the effects it has had on our mental health. The consequent quarantine and isolation have been connected with fear (Rubin and Wessely, 2020), acute stress syndrome, depression, post-traumatic stress disorder, insomnia, irritability, anger, emotional exhaustion, and a perceived loss of control (Jung and Jun, 2020; Xiao, 2020). In their review, Brooks et al. (2020) noted that the most frequently reported negative effects of lockdown were post-traumatic stress disorder, confusion, and anger. If these effects were true for the general population, we can make the assumption that they were amplified in health workers by three main factors (Tarquinio et al., 2020): social pressure due to the rapid evolution of the emergency situation and the consequent changes in the quality of care they could provide; professional pressure due to a far higher than normal work load and personal pressure due to the fear of transmitting the virus to their loved ones or the distress of being isolated.

This assumption is even more fitting for staff operating in residential nursing homes for the elderly (residenze sanitarie assistite [RSA]). In Italy, RSAs refer to residential communities which accommodate elderly, weak or vulnerable people with multiple assistance needs: physical and medical, emotional, and relational. It was in these places that the sudden COVID-19 pandemic had its most catastrophic impact.

A survey of how the virus spread during the first wave of the Pandemic (Lombardo et al., 2021), covering about 40% of Italian RSAs, showed that 9,154 residents deceased from 1 February to 5 May 2020.

In the same period, 12% of the nursing homes had at least one COVID-positive resident, 35% had at least one resident with “flu-like symptoms” and 21% had at least one COVID-positive health worker. The authors point out that 60% of nursing homes received no precise indications on how to manage the residents or on how to prevent or handle infections. About 8% of nursing homes were unable to isolate residents with suspected or confirmed COVID-19, and the arrival of the Pandemic highlighted critical problems like the shortage of personnel, the difficulty of transferring patients from one structure to another, the lack of personal protective equipment (PPE) and medicines, and the fact that it was impossible to conduct COVID-19 PCR test (molecolar, swab) when necessary. This situation with so many critical issues may have generated a number of stressors with negative effects on the mental health of nurses and other staff, as has already been observed for other major disasters and pandemics (Alharbi et al., 2020; Usher et al., 2020).

During the first wave of the COVID-19 pandemic, the Italian EMDR association (Associazione EMDR Italia) was called on by nursing home directors to organize a cycle of EMDR sessions to support staff through this challenging period. The Eye Movement Desensitization and Reprocessing-Integrative Group Treatment Protocol (EMDR-IGTP) has already been reported to be effective in supporting staff involved in dealing with emergency situations (Tarquinio et al., 2020) and it is suitable to be delivered online (Perri et al., 2021). Recently, however, Lenferink et al. (2020) noted that there is a lack of empirical data to confirm its successfulness in the treatment of post-traumatic stress disorder due to the COVID-19 Pandemic.

We think that our report partially fills this gap by fulfilling two objectives:

• it provides a current picture of the state of psychological health of a group of staff in nursing homes during the first wave of the COVID-19 pandemic; and
• it supplies quantitative data on the effectiveness of the online group EMDR treatment on this hard-hit population.

## Research context

The online group EMDR treatment was administered to health care, social, and administrative staff or workers in 6 RSAs throughout Italy. An agreement with the *Associazione EMDR Italia* was reached and signed by each facility. Twenty-six qualified EMDR therapists volunteered to participate in the project.

The aim of the project was to provide the nursing homes workers involved with support to bear the emotional load caused by isolation, to reduce stress and to contain states of anxiety and anger. Also to work through negative feelings and traumatic moments in order to avoid the development of problems related to post-traumatic stress.

The following nursing homes participated in the project:

• RSA Villa San Lorenzo (Trentino Alto Adige): 49 workers divided into in 17 groups;
• RSA Rosa dei Venti (Trentino Alto Adige): 47 workers divided into in 17 groups;
• RSA Padre Odone Nicolini (Trentino Alto Adige): 37 workers divided into 13 groups;
• RSA Telegonia (Sardegna): 11 workers divided into 3 groups;
• RSA Centro Monsignore Siro Silvestri (Liguria): 26 workers divided into 5 groups;
• RSA Centro socio assistenziale del Sacro Cuore (Liguria): 14 workers divided into 3 groups.

## Materials and methods

A total of 58 interventions were carried out, each consisting of a minimum of 3 and a maximum of 5 sessions, with groups of 2–5 participants. Each intervention was conducted by an specifically trained, qualified EMDR therapist external to the nursing home. All the meetings were conducted on a digital conferencing platform to guarantee social distancing. All the interventions were carried out during one of the acute phases of the emergency (spring–summer 2020).

In the first meeting participants were given psychoeducational instruction on the reactions caused by post-traumatic stress disorder in accordance with the CISO model (*Critical Incident Stress Orientation*) (Maslovaric, 2020), which involves:

• giving participants a symptom grid showing normal and common reactions to post-traumatic stress. This allows participants to identify their own symptoms as common, albeit disturbing. It also provides explanations and descriptions of what is meant by a traumatic event, the psychological reactions that can arise following one, and vulnerability factors (in this case level of involvement and exposure);
• providing indications for emotional self-protection to promote coping strategies and resilience;
• providing explanations and an introduction to EMDR therapy and in particular on the brief group protocol (EMDR-IGTP).

This was followed by brief defusing and debriefing interventions to stabilize participants and inform them about acute stress and post-traumatic reactions and to strengthen individual coping resources. Finally, the safe place was established by means of the butterfly hug technique (Artigas and Jarero, 2014; Jarero, 2020).

The butterfly hug technique used in the group protocol (EMDR-IGTP), is a form of self-administered bilateral stimulation used to achieve stabilization, to enhance resources and to create a safe place; and to re-process traumatic events (Artigas and Jarero, 2014; Jarero, 2020).

Following this initial preparatory phase, the main body of the treatment consisted of applying the EMDR-IGTP (Jarero and Artigas, 2009) to reprocess the main traumatic events identified by the participants. These events included being powerless to help elderly patients who were in pain and unable to breathe; watching the death of patients with whom they had had strong emotional bonds; being unable to respond to sick patients’ requests for a hug; being unable to take adequate care of the bodies of deceased patients; the fear of transmitting the disease to patients or to their own families; the lack of personal protective equipment and the fear of legal liability for the high number of deaths (in the case of participants in roles with legal responsibility). The following are some of the testimonies collected during the intervention:

• *“I cried for the whole shift and I couldn’t stop even when I got home*”;
• “*Watching them die and not being able to touch them because I was scared of getting infected*”;
• *“Watching the old people getting worse one by one”;*
• *“Rushing from one room to the other without really being able to do anything”;*
• “*Not enough space in the morgue for the bodies”;*
• *“Not being able to get away from it.”*

These perceptions (and others in the paragraph on the participants’ experiences) were the target traumatic experiences and events for EMDR treatment during the interventions.

## Outcome measurements

The assessment protocol was based on two self-report questionnaires.

The characteristics of the sample were studied through *ad hoc* questions: (1) socio-demographic (age, sex, number of children, number of cohabitants); (2) job-related information (e.g., workplace and occupation). The post-traumatic symptomatology was evaluated through Impact of Event Scale-Revised (IES-R) in accordance with the criteria of the DSM IV-TR (Weiss and Marmar, 1997) validated and translated into Italian (Pietrantonio et al., 2003). The latest PCL-5 (American Psychiatric Association, 2013) was not used, as it is free (in Italian version) as of May 2019. We considered it late and for the reasons of homogeneity we always did the IES-R. This psychometric test consists of 22 items. It includes 3 subscales measuring the following dimensions: intrusion, avoidance, and hyperactivation. Participants were asked to rate their level of post-traumatic symptoms using a 5-point Likert scale ranging from 0 (=“not at all”) to 4 (=“a lot”) referring to the previous 7 days. The total score between 0 and 88. The cut-off of 33 highlights a high risk of PTSD; in line with the literature, there are no specific cut-offs for scale interpretations. The Italian translation of IES-R has shown satisfactory internal validity in studies on different populations at risk, as reported by Craparo et al. (2013) (Intrusion, α = 0.78; Avoidance, α = 0.72; Hyperarousal, α = 0.83) and Converso and Viotti (2014) (Intrusion, α = 0.91; Avoidance, α = 0, 81; Hyperarousal, α = 0.87). Although the IES-R has not been validated in the general Italian population, it has been used to evaluate the symptomatology of PTSD in many Italian samples, which confirmed its adequate reliability (Gambetti et al., 2011; Priebe et al., 2011; Maslovaric et al., 2017).

The Emotion Thermometer (THERMO, Mitchell et al., 2010), a visual analog self-assessment scale to collect the level of intensity of emotional activation on a Likert scale from 1 to 10 regarding some main emotional experiences (stress, depressed mood, anxiety, anger, sleep problems, need for help) during the previous week was also submitted to the investigated subjects.

These questionnaires were administered by psychologists different to the ones who carried out the group EMDR interventions.

### Respondents

The project involved 184 subjects. Data are reported for the 122 who completed both the pre- and post-treatment questionnaires. The differential attrition in this study was less than 15%. This sample comprised 102 females and 20 males with an average age of 44.

### Participants’ experiences

• Working in a ward turned upside down by the emergency (rushing from one room to another without really knowing what to do, having to cover staff shortages by working shifts of up to 12 h);
• the tragic deaths of many of the nursing home guests and witnessing their suffering without being able to touch them (patients struggling to breathe and dying);
• not being able to carry out the normal passing rituals associated to death, due to the high numbers of deceased patients;
• fear of infection for themselves and their families; the sense of guilt over having infected someone; social distancing from their own families;
• coincidence of the COVID-19 emergency with personal traumas (like cancer in a family member).

The distress of these experiences was accompanied by workers’ negative beliefs about their own ability to deal with the traumatic situation (I don’t trust myself, I’m not in control, I’m helpless, I’m in danger, I’m useless, I’m unable to handle this); and by somatic symptoms like sleepiness/difficulty sleeping, anxiety/worry and irritability/restlessness.

The aim of this group EMDR treatment (Jarero and Artigas, 2009) was to enhance positive emotions and internal resources by installing coping strategies and resilience. The following resources were to be reinforced; the sense of belonging to a working group and to a team (humanity, unity, the humbleness to accept help; collaboration, sharing, closeness, solidarity and support), a sense of control in situations where control was possible, a sense of self effectiveness, the ability to appreciate life.

## Results

The following Table 1 reports the descriptive statistics for all the analyzed variables, both as such (i.e., subdivided in the pre and post conditions) and, much more relevant for the paired character of the experimental plan, the pre-post difference (deltavariables).

**TABLE 1:** Descriptive statistics of variables analyzed.

Variable	*N*	Mean	Standard deviation
Sex	122	0.1639344	0.3717427
Age	122	44.6311475	10.6337999
IES_Avoidance_PRE	122	10.8032787	6.2663200
IES_Intrusivenss__PRE	122	12.7868852	6.7996469
IES_Hyperarousal_PRE	122	9.1229508	5.8980006
IES_TOTAL_PRE	122	32.7131148	16.8696899
IES_Avoidance_POST	122	8.0491803	5.9887670
IES_Intrusivenss__POST	122	7.7377049	6.3059768
IES_Hyperarousal_POST	122	5.4508197	5.2050555
IES_TOTAL_POST	122	21.2377049	16.2867148
THERMO__PRE_STRESS	122	5.3565574	2.6162868
THERMO__PRE_ANXIETY	122	4.6229508	3.1177388
THERMO__PRE_MOOD	122	3.0286885	2.7740676
THERMO__PRE_ANGER	122	3.6967213	3.1317868
THERMO__PRE_SLEEP	122	3.9180328	3.3865074
THERMO__PRE_AHELP	122	4.0371901	2.8063657
THERMO__POST_STRESS	122	4.0204918	2.5417160
THERMO__POST_ANX	122	3.1598361	2.7342837
THERMO__POST_MOOD	122	2.1024590	2.3905276
THERMO__POST_ANGER	122	2.3934426	2.3795179
THERMO__POST_SLEEP	122	2.3442623	2.6403434
THERMO__POST_HELP	122	2.2581967	2.2528527

Together with the observables, we report two demographic variables, i.e., sex and age. Given males are coded by 1 and female by 0, the mean of this variable corresponds to the proportion of males in the population that is equal to 0.16, the highly unbalanced gender composition of the data set prevents to use this variable as covariate. The age ranges from 19 to 62 years old and has a mean value of around 45 years (M = 44.63; SD = 10.63).

The paired character of the variables prompted us to adopt a paired-test strategy, correspondent to a one sample test on the null hypothesis of delta variables = 0 (identity of pre and post condition) (Table 2). The inferential tests were of three types: (1) a parametric test (Student’s *t*-test, T), (2) a non-parametric binary test on the (sign of the difference, M), and (3) a non-parametric (Mann–Whiney U on ranks, S).

**TABLE 2:** Statistical variables.

	*N*	Mean	SD	Median	Min	Max
deltaavoidance	122	2.75410	6.43250	2.00000	−17.00000	18.00000
deltaintrusiveness	122	5.04918	7.20061	4.00000	−15.00000	25.00000
deltahyperarousal	122	1.38525	6.34341	1.00000	−17.00000	16.00000
deltaiestotal	122	11.47541	17.10648	9.50000	−31.00000	54.00000
deltastress	122	1.33607	2.85203	1.00000	−5.00000	8.00000
deltanxiety	122	1.46311	2.67771	1.00000	−6.00000	8.00000
deltamood	122	0.92623	2.69041	0	−8.00000	8.00000
deltaanger	122	1.59426	3.23356	1.00000	−8.00000	9.00000
deltasleep	122	1.57377	2.73610	1.00000	−7.00000	10.00000
deltahelp	121	1.78512	2.67537	2.00000	−6.00000	9.00000

Here below the results showing a marked significance for all the test for all the observed variables with only one exception for deltahyperarousal sign test (Table 3).

**TABLE 3:** Inferential tests.

	Mu0 = 0				
Variable	Test		Statistical	*P*-value	
deltaavoidance	Student’s *t*-test	t	4.729.112	Pr > | t|	<0.0001
	Sign	M	22	Pr >= | M|	<0.0001
	Mann–Whiney *U*	S	1624.5	Pr >= | S|	<0.0001
deltaintrusiveness	Student’s *t*-test	t	7.745.179	Pr > | t|	<0.0001
	Sign	M	34	Pr >= | M|	<0.0001
	Mann–Whiney *U*	S	2401	Pr >= | S|	<0.0001
deltahyperarousal	Student’s *t*-test	t	2.412.039	Pr > | t|	0.0174
	Sign	M	9.5	Pr >= | M|	0.0928
	Mann–Whiney *U*	S	778	Pr >= | S|	0.0290
deltaiestotal	Student’s *t*-test	t	7.409.476	Pr > | t|	<0.0001
	Sign	M	35	Pr >= | M|	<0.0001
	Mann–Whiney *U*	S	2491	Pr >= | S|	<0.0001
deltastress	Student’s *t*-test	t	5.174.328	Pr > | t|	<0.0001
	Sign	M	20.5	Pr >= | M|	<0.0001
	Mann–Whiney *U*	S	1407.5	Pr >= | S|	<0.0001
deltanxiety	Student’s *t*-test	t	6.035.249	Pr > | t|	<0.0001
	Sign	M	27	Pr >= | M|	<0.0001
	Mann–Whiney *U*	S	1622	Pr >= | S|	<0.0001
deltamood	Student’s *t*-test	t	3.802.593	Pr > | t|	0.0002
	Sign	M	12.5	Pr >= | M|	0.0097
	Mann–Whiney *U*	S	903	Pr >= | S|	<0.0001
deltaanger	Student’s *t*-test	t	5.445.762	Pr > | t|	<0.0001
	Sign	M	19.5	Pr >= | M|	0.0001
	Mann–Whiney *U*	S	1411	Pr >= | S|	<0.0001
deltasleep	Student’s *t*-test	t	6.353.151	Pr > | t|	<0.0001
	Sign	M	25.5	Pr >= | M|	<0.0001
	Mann–Whiney *U*	S	1429.5	Pr >= | S|	<0.0001
deltahelp	Student’s *t*-test	t	7.339.679	Pr > | t|	<0.0001
	Sign	M	29	Pr >= | M|	<0.0001
	Mann–Whiney *U*	S	1710	Pr >= | S|	<0.0001

The statistical significance of all the differential variables comes from the strong pairwise correlation between the different test here reported in terms of Spearman correlation coefficient (Table 4). It is worth noting the stronger correlation existing among tests of the same battery (IES and Emotion Thermometer) with respect to tests pertaining to different batteries.

**TABLE 4:** Spearman correlation coefficient.

	deltaavoidance	deltaintrusiveness	deltahyperarousal	deltaiestota	deltastress	deltanxiety	deltamood	deltaanger	deltasleep	deltahelp
deltaavoidance	1.00000	0.58713	0.57086	0.80406	0.189	0.130	0.0900	0.1304	0.2155	0.107
		<0.0001	<0.0001	<0.0001	0.036	0.152	0.3241	0.1522	0.0171	0.240
	122	122	122	122	122	122	122	122	122	121
deltaintrusiveness	0.58713	1.00000	0.84394	0.91240	0.207	0.187	0.1848	0.3397	0.3185	0.177
	<0.0001		<0.0001	<0.0001	0.022	0.038	0.0415	0.0001	0.0003	0.051
	122	122	122	122	122	122	122	122	122	121
deltahyperarousal	0.57086	0.84394	1.00000	0.85978	0.223	0.193	0.2089	0.3965	0.2482	0.147
	<0.0001	<0.0001		<0.0001	0.0135	0.0329	0.0209	<0.0001	0.0058	0.1062
	122	122	122	122	122	122	122	122	122	121
deltaiestotal	0.80406	0.91240	0.85978	1.00000	0.275	0.228	0.2003	0.3374	0.3375	0.213
	<0.0001	<0.0001	<0.0001		0.002	0.011	0.0269	0.0001	0.0001	0.018
	122	122	122	122	122	122	122	122	122	121
deltastress	0.18913	0.20718	0.22302	0.27531	1.000	0.772	0.5555	0.5274	0.4736	0.472
	0.0369	0.0220	0.0135	0.0021		<0.0001	<0.0001	<0.0001	<0.0001	<0.000
	122	122	122	122	122	122	122	122	122	121
deltanxiety	0.13031	0.18754	0.19328	0.22816	0.772	1.000	0.6243	0.5089	0.4061	0.513
	0.1526	0.0386	0.0329	0.0115	<0.0001		<0.0001	<0.0001	<0.0001	<0.000
	122	122	122	122	122	122	122	122	122	121
deltaumood	0.09002	0.18487	0.20895	0.20033	0.555	0.624	1.0000	0.5771	0.3793	0.448
	0.3241	0.0415	0.0209	0.0269	<0.0001	<0.000		<0.0001	<0.0001	<0.000
	122	122	122	122	122	122	122	122	122	121
deltaanger	0.13041	0.33977	0.39658	0.33749	0.527	0.50	0.5771	1.0000	0.2254	0.441
	0.1522	0.0001	<0.0001	0.0001	<0.0001	<0.00	<0.0001		0.0125	<0.000
	122	122	122	122	122	122	122	122	122	121
deltasleep	0.21558	0.31852	0.24824	0.33751	0.473	0.40	0.3793	0.2254	1.0000	0.322
	0.0171	0.0003	0.0058	0.0001	<0.0001	<0.00	<0.0001	0.0125		0.000
	122	122	122	122	122	122	122	122	122	121
deltahelp	0.10757	0.17761	0.14759	0.21309	0.472	0.51	0.4484	0.4415	0.3222	1.00
	0.2402	0.0513	0.1062	0.0189	<0.0001	<0.00	<0.0001	<0.0001	0.0003	
	121	121	121	121	121	121	121	121	121	121

In any case all the correlation coefficient (both intra- and inter- batteries) were highly statistically significant, this gave rise to a very clear principal component structure, with a leading component (PC1) reporting the “consensus” among all the tests with all positive loadings, a clear “shape” component pointing to the difference among IES and Thermometer variables (that have opposite loading sign on PC2), and a minor third component (PC3) pointing to an opposition between the magnitude on the effect on anger and on sleep.

PC1 accounts for almost the half of entire variance (45%) while PC2 and PC3 account for 24% and 8% of total variance so pointing to a global consistency of the results of different tests that collapse into a “consensus” (PC1) major component (Tables 5, 6).

**TABLE 5:** Eigenvalues of the correlation matrix: Total = 10 Mean = 1.

	Eigenvalue	Difference	Proportion	Cumulative
1	4.52693273	2.08378055	0.4527	0.4527
2	2.44315218	1.64263147	0.2443	0.6970
3	0.80052071	0.15599193	0.0801	0.7771
4	0.64452878	0.09131861	0.0645	0.8415
5	0.55321017	0.17980396	0.0553	0.8968
6	0.37340621	0.08551461	0.0373	0.9342
7	0.28789160	0.06847095	0.0288	0.9630
8	0.21942065	0.08439755	0.0219	0.9849
9	0.13502310	0.11910921	0.0135	0.9984
10	0.01591389		0.0016	1.0000

**TABLE 6:** Loading pattern.

	PC1	PC2	PC3
deltaavoidance	0.59898	–0.58646	0.21103
deltaintrusiveness	0.76229	–0.53916	–0.09758
deltahyperarousal	0.73656	–0.53417	–0.17753
deltaiestotal	0.79636	–0.58924	0.01524
deltastress	0.68855	0.48597	0.24739
deltanxiety	0.66312	0.54209	0.17247
deltamood	0.62445	0.53589	–0.17941
deltaanger	0.68201	0.33747	–0.49469
deltasleep	0.59928	0.21619	0.55662
deltahelp	0.53134	0.44513	–0.19246

The PC1 scores were used to detect (if any) an effect of covariates on the global amelioration of subjects (PC1). No covariate (sex, age, center, having had COVID) gave rise to a statistical significance at Analysis of variance. It is very important the lack of a statistical significant effect of “center” covariate. This result points to the feasibility of a general analysis encompassing all the different centers.

## Discussion

Our study provides a snapshot of one of the populations most exposed to physical and emotional distress during the most critical time of the COVID-19 pandemic. The picture is one of long shifts and a sense of complete exhaustion, no time to drink or eat, rushing from one patient to another without really being able to help and feeling helpless themselves.

Eye movement desensitization and reprocessing treatments helped re-process traumas experienced during the emergency by giving participants somewhere share what was happening to them. The prompt timing of the treatment enabled participants to recover their capacity to face the prolonged stress and may have prevented the consequent problems from becoming chronic and leaving permanent scars. The EMDR meetings also had the important benefit of joining into work groups and providing them with a space where participants could express their emotions and re-process the most difficult moments together, creating a greater sense of unity and strength.

Consistently with this description, the IES-R (Weiss and Marmar, 1997) scores obtained by participants following the treatment phase of the treatment were significantly lower on the intrusiveness, avoidance and arousal scales. The percentage of participants with a score above the PTSD threshold went down from 35.1 to 18.85%. The pre- and post-treatment emotion thermometer scores also showed a significant decrease in levels of stress, anxiety, depressed mood, anger, difficulty sleeping, and perceived need for help. These differences show that our online EMDR treatment was successful in reducing PTSD symptoms and lead to increased wellbeing of the participants.

Health workers involved in responding to the emergency, whether in clinical or community settings, are key to the success of our battle against the pandemic. As much effort as possible should be invested in protecting their mental and physical health: psychological support, both during and following the emergency period can enable them to adapt and empower them individually and collectively.

The events witnessed during the first wave of the pandemic showed that nursing homes staff could benefit from EMDR treatments beyond the acute phase of the pandemic. This backs up the importance of conducting further research into the effectiveness of EMDR conducted online, as asserted by Lenferink et al. (2020) in their review. Although the study they reviewed reported good results for internet-delivered EMDR for PTSD, it lacked a control group and the only criterion for inclusion was the patient’s need for psychological support.

Group EMDR seems to be a useful instrument for both prevention and treatment. Our results are in line with those of other studies in showing that the standard EMDR protocol can be successfully adapted for use online to conduct EMDR therapy online (Fisher, 2021), including on workers involved in healthcare during the COVID-19 emergency (Tarquinio et al., 2020).

In conclusion, future research involving larger samples and control groups is necessary to properly assess the effectiveness of group psychotherapy conducted online.

COVID-19 had a significant impact on the wellbeing of healthcare workers that need for mental health protection, support, and treatment. This study demonstrated that interventions with EMDR for this population had a positive effect to significantly decrease symptoms such as stress, depressed mood, anxiety, anger, sleep problems, and need for help.

This confirms that working with EMDR in emergency situations provokes immediate relief, prevents chronicization.

Also, the study confirmed that EMDR protocol in online modalities could protect healthcare workers from the consequences of acute stress.

In conclusion, the possibility in the future of collecting further data may improve the statistical strength of the study and observe the resilience of a specific population as time goes on, in order to understand if an early intervention with EMDR, during a critical event, can help the growth of this evolutionary skill.

## Limitations

The emergency situation did not give the possibility to deepen further aspects that would have been important for the research, however it was possible to have a not treatment group, in order to understand the effectiveness of the EMDR protocol on a specific population.

Although the results of the present study are encouraging several limitations are present.

A limitation is represented by the use and analysis of only two standardized tests. In addition, other psychopathologies were not investigated as an outcome measure. The administration of other psychometric tests for the assessment of other psychopathologies may be functional for future research.

Moreover, the efficacy of EMDR treatment was evaluated in only two times (pre-post) without a possibility of *follow up* and, therefore, the absence of a longitudinal control aimed at following the reduction of PTSD symptoms over time.

Certainly, these limitations reduce the generalization of the results and may have affected the study.

## Data availability statement

The raw data supporting the conclusions of this article will be made available by the authors, without undue reservation.

## Ethics statement

Ethical review and approval was not required for the study on human participants in accordance with the local legislation and institutional requirements. The patients/participants provided their written informed consent to participate in this study.

## Author contributions

GiM, EF, MG, GaM, LP, SR, AI, AN, and MP contributed to the conception and design of the study. MG, GiM, LP, SR, AI, and AN organized the database. MP performed the statistical analysis. All authors wrote the first draft and sections of the manuscript, contributed to manuscript revision, read, and approved the submitted version.
